# Supplementary material for: Endogenous renal adiponectin drives gluconeogenesis through enhancing pyruvate and fatty acid utilization
Source: Nat Commun. 2023 Oct 17;14:6531. doi: 10.1038/s41467-023-42188-4 (PMC10582045; doi:10.1038/s41467-023-42188-4)
Supplement: Supplementary file 1 — Supplementary Information [file 41467_2023_42188_MOESM1_ESM.pdf]

Supplementary Information

Endogenous renal adiponectin drives gluconeogenesis through enhancing pyruvate and fatty acid utilization.

Toshiharu Onodera [a], May-Yun Wang [a], Joseph M Rutkowski [b], Stanislaw Deja [c], Shiuhwei Chen [a], Michael S Balzer [d], Dae-seok Kim [a], Xuenan Sun [a], Yu A An [a], Bianca Field [a], Charlotte Lee [e], Ei-ichi Matsuo [f], Monika Mizerska [c], Ina Sanjana [f], Naoto Fujiwara [g], Christine M Kusminski [a], Ruth Gordillo [a], Laurent Gautron [e], Denise K Marciano [h], Ming Chang Hu [i, j], Shawn Burgess [c], Katalin Susztak [d], Orson W Moe [i, j, k], Philipp E Scherer [a, h]

[a] Touchstone Diabetes Center, Department of Internal Medicine, The University of Texas Southwestern Medical Center, Dallas, United States.
[b] Division of Lymphatic Biology, Department of Medical Physiology, Texas A&M University College of Medicine, Bryan, Texas, USA.
[c] Center for Human Nutrition, University of Texas Southwestern Medical Center, Dallas, TX, United States.
[d] Renal, Electrolyte, and Hypertension Division, Department of Medicine, University of Pennsylvania Perelman School of Medicine, Philadelphia, PA 19104, USA.
[e] Center for Hypothalamic Research, University of Texas Southwestern Medical Center, Dallas, TX, USA.
[f] Solutions COE, Analytical & Measuring Instruments Division, Shimadzu Corporation, Kyoto, Japan
[g] Liver Tumor Translational Research Program, Simmons Comprehensive Cancer Center, Division of Digestive and Liver Diseases, Department of Internal Medicine, University of Texas Southwestern Medical Center, 5323 Harry Hines Blvd, Dallas, TX 75390, USA.
[h] Departments of Cell Biology and Internal Medicine, University of Texas Southwestern Medical Center, Dallas, TX, USA.
[i] Charles and Jane Pak Center for Mineral Metabolism and Clinical Research, University of Texas Southwestern Medical Center, Dallas, Texas, USA.
[j] Department of Internal Medicine, University of Texas Southwestern Medical Center, Dallas, Texas, USA.
[k] Department of Physiology, University of Texas Southwestern Medical Center, Dallas, Texas, USA.

Corresponding author: Philipp E Scherer
Touchstone Diabetes Center, Department of Internal Medicine, The University of Texas Southwestern Medical Center, Dallas, United States.
e-mail: Philipp.Scherer@UTSouthwestern.edu
Tel: +1-214-648-8715 Fax: (214) 648-8720

List of Supplement Materials

1. Supplementary Figures S1 to S10

2. Tables S1 and 2

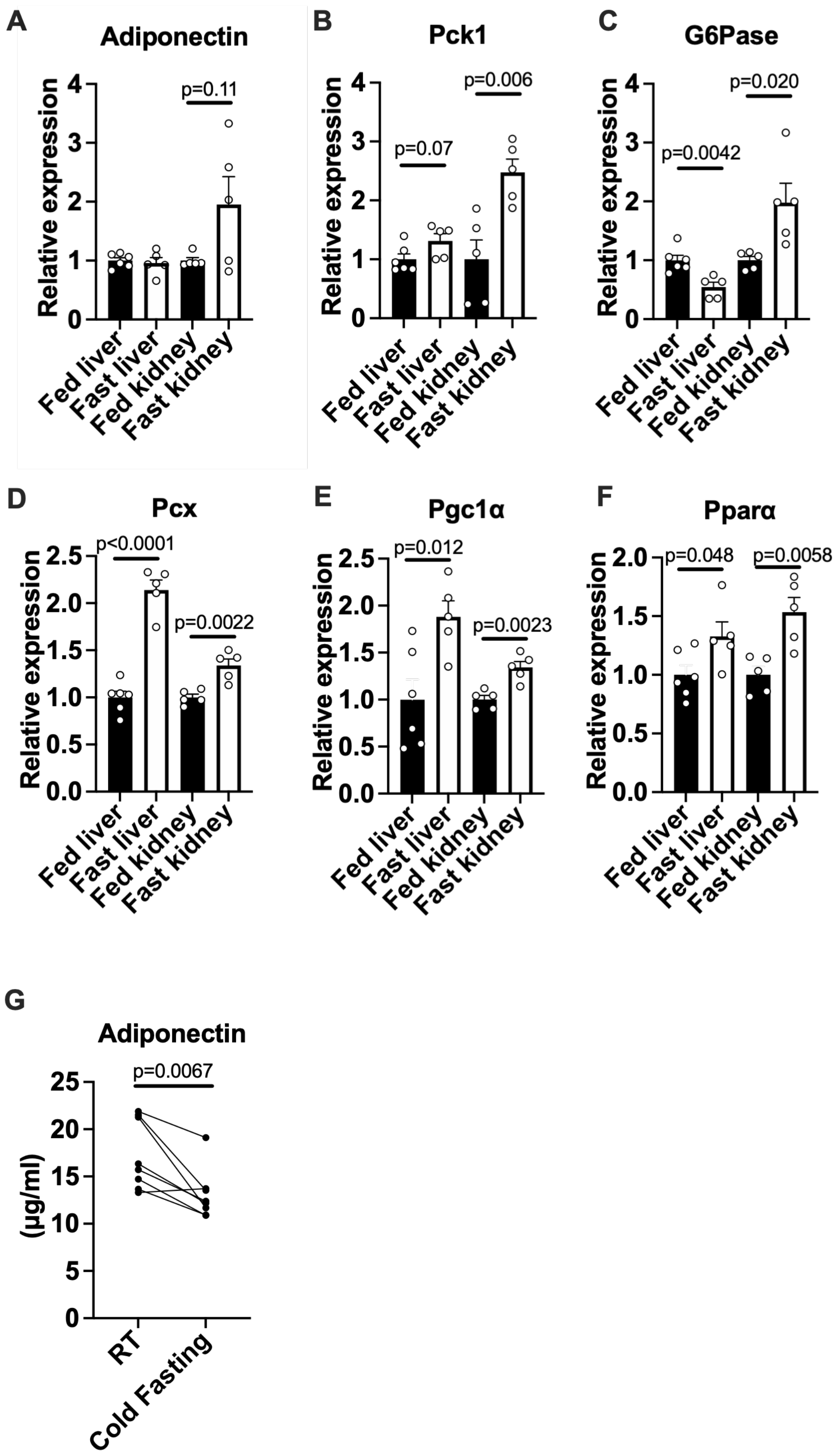

Figure S1. *Gluconeogenesis related gene expressions in liver and kidney under fed and 24-hour fasting conditions.*
(A-F) Gluconeogenesis related gene expressions including Adiponectin, *Pck1*, *G6Pase*, *Pcx*, *Pgc1α*, and *Pparα* in liver and kidney were determined under fed and

24-hour fasting conditions. (Fed liver n=6, Fast liver n=5, Fed kidney n=5, Fast kidney n=5) **(G)** Serum adiponectin levels before and after cold exposure and fasting. (n=8) Data are mean ±SEM. Unpaired two-tailed t-tests were performed for statistics from (**A**) to (**G**).

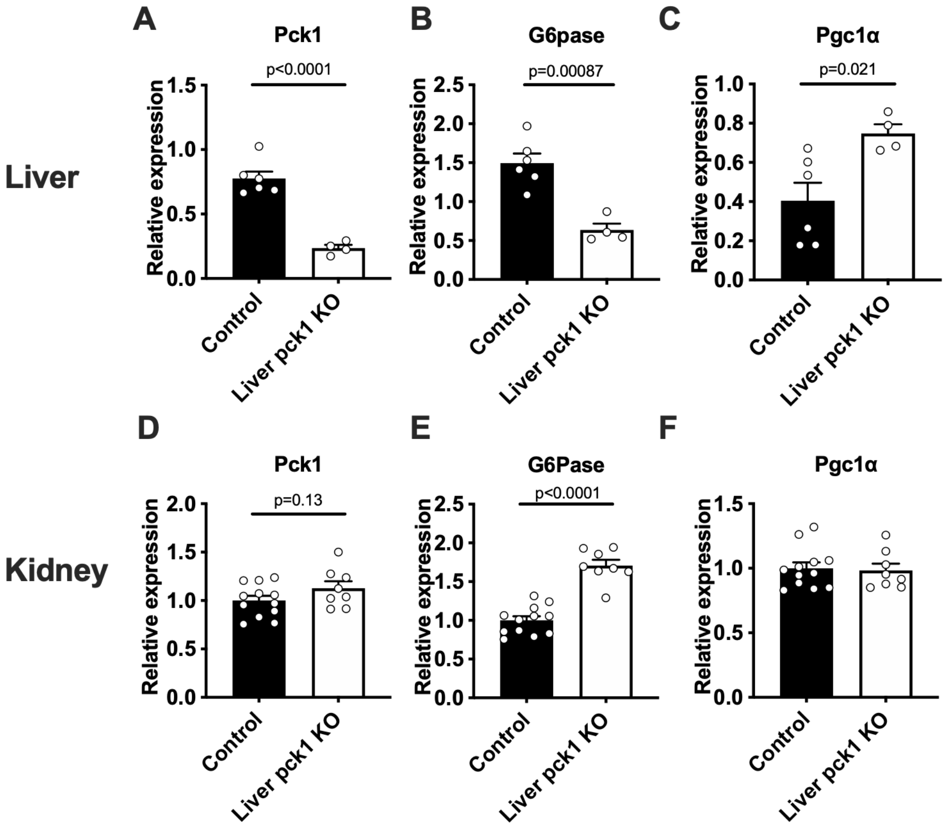

Figure S2. *Gluconeogenesis related gene expressions in liver specific Pck1 KO mice*

(A-C) Gluconeogenesis related gene expressions in the liver of liver specific Pck1 KO mice (Control n=6, Liver pck KO n=4).

(D-F) Gluconeogenesis related gene expressions in the kidney of liver specific Pck1 KO mice. Left and right kidneys were utilized for this analysis (Control n=12, Liver pck KO n=8). Data are mean ±SEM. Unpaired two-tailed t-tests were performed for statistics from (**A**) to (**F**).

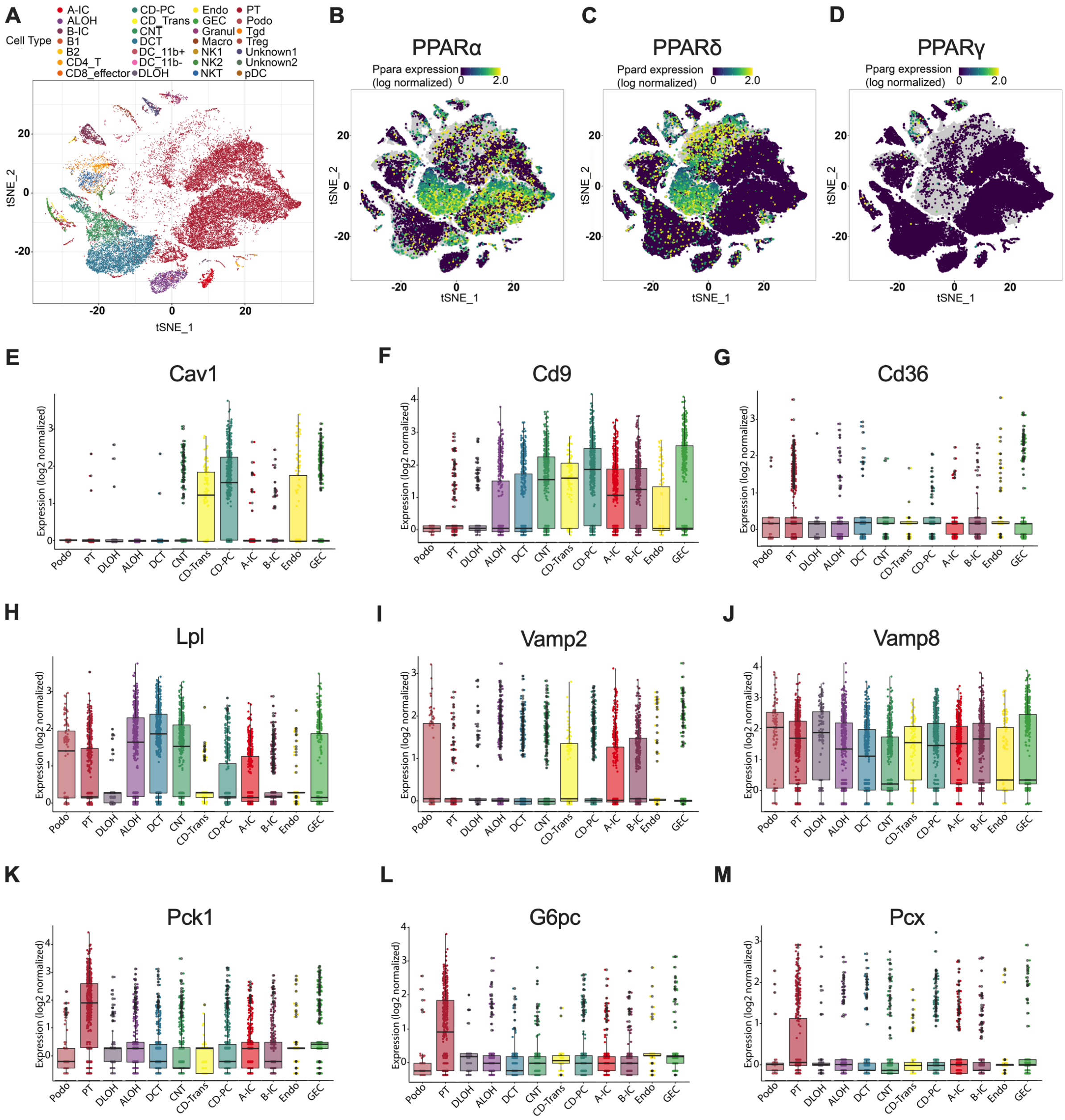

Figure S3. *Single cell based renal gene expressions related to lipid metabolism and gluconeogenesis*

the tSNE (t-distributed stochastic neighbor embedding) plot was generated for the entire dataset of single cell RNA-seq of kidney cells.

(A) The distribution of 28 distinct cell populations in the tSNE plot.

(B-D) PPARs gene expression feature plot for the entire kidney cells.

(E-J) Single cell RNA-seq expressions of lipid metabolism related genes in log2 scale. Assigned cell types are described as follows. Podo, podocyte; PT, proximal tubule; DLOH, descending loop of Henle; ALOH, ascending loop of Henle; DCT, distal convoluted tubule; CNT, connecting tubule; CD-Trans, collecting duct transitional cell; CD-PC, collecting duct principal cell; A-IC, alpha intercalated cell; B-IC, beta intercalated cell; Endo, endothelial; GEC, glomerular endothelial cells

(K-M) Single cell RNA-seq expressions of gluconeogenesis related genes in log2 scale.

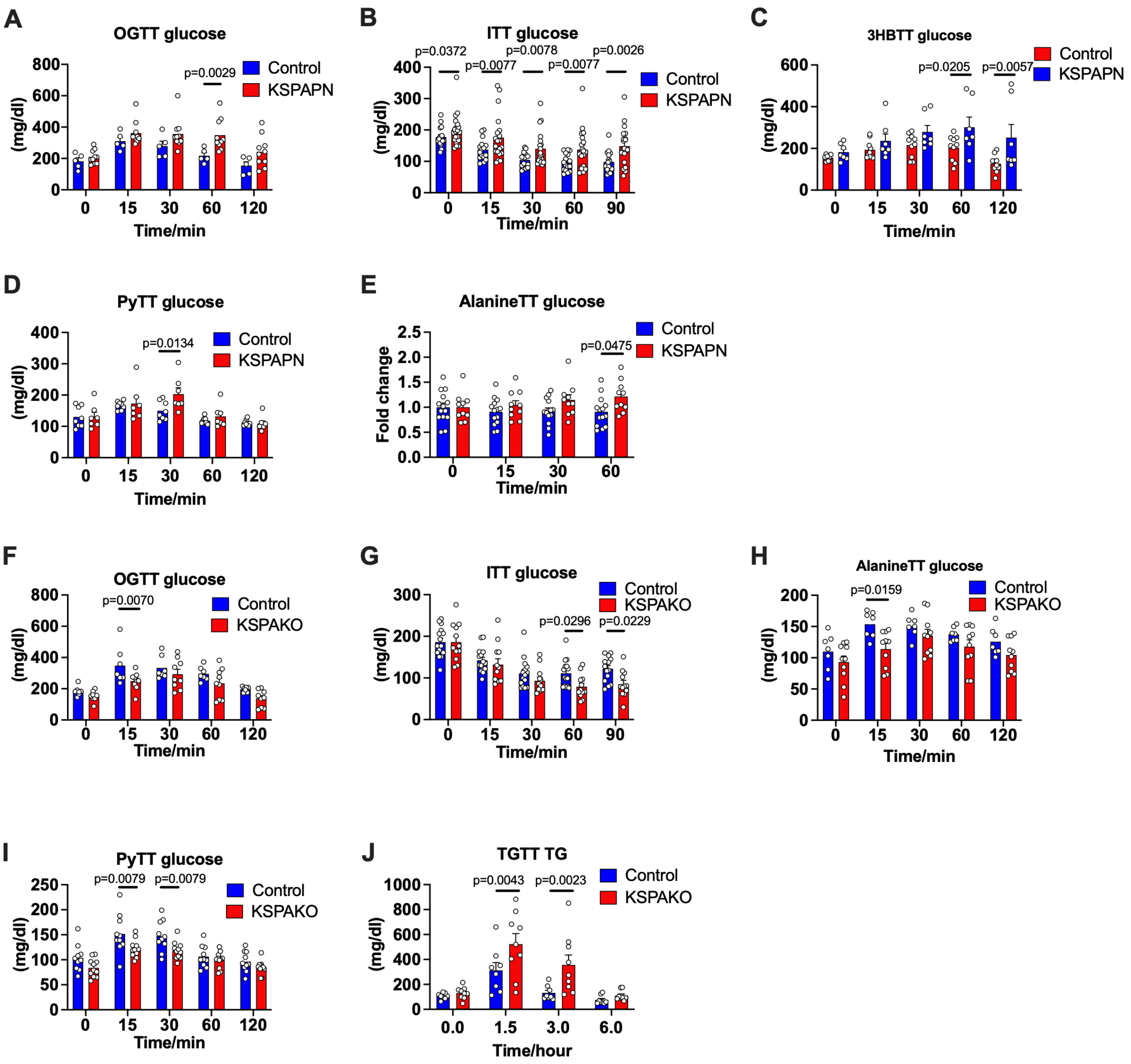

Figure S4. *Individual data points of tolerance test in KSPAPN and KSPAKO cohorts* (**A**) Blood Glucose levels at different time points in OGTT (Control n=5, KSPAPN n=10) (**B**) Blood glucose level at different time points after insulin injection (Control n=16, KSPAPN n=20) (**C**) Blood glucose levels at different time points after 3HB injection (Control n=10, KSPAPN n=7). (**D**) Blood glucose levels at different time points after pyruvate gavage (Control n=8, KSPAPN n=7). (**E**) Blood glucose levels (fold-change) at different time points after alanine injection (Control n=14, KSPAPN n=10). (**F**) Blood Glucose levels at different time points during an OGTT (Control n=7, KSPAKO n=9). (**G**) Blood Glucose levels at different time points in ITT (Control n=15, KSPAKO n=12). (**H)** Blood glucose levels at different time points during an alanine tolerance test (Control n=7, KSPAKO n=10). (**I**) Blood glucose levels at different time points during a pyruvate tolerance test (Control n=10, KSPAKO n=11). (**J**) Blood TG levels at different time points during a TG clearance test (Control n=8, KSPAKO n=9). Data are mean ±SEM. 2-way ANOVA with 2-stage linear step-up procedure of BKY correction for multiple comparisons were performed to determine p-values from (**A**) to (**J**).

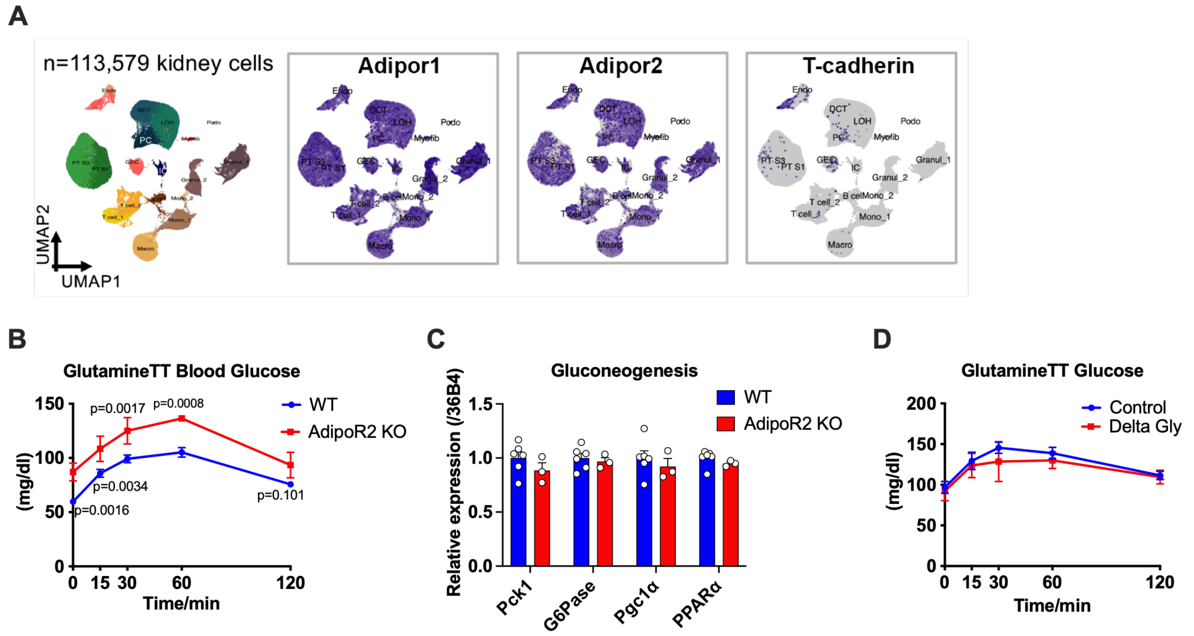

Figure S5. *Expressions of AdipoRs and T-cadherin in the kidney and the contribution of AdipoR2 to gluconeogenesis*

(**A**) UMAP projection of 113579 kidney cells. Gene expression feature plots of kidney cells projected onto UMAP. Adipor1, Adipor2 and T-cadherin. (**B**) Blood glucose level during glutamine tolerance test in global AdipoR2 KO mice under normal chow feeding (Control n=8, AdipoR2 KO n=5). 2-way ANOVA with 2-stage linear step-up procedure of BKY correction for multiple comparisons were performed to determine p-values. (**C**)Expression of genes involved in gluconeogenesis in global AdipoR2 KO kidney under HFD condition (Control n=6, AdipoR2 KO n=3). (**D**) Blood glucose levels during a glutamine tolerance test in *ΔGly* adiponectin overexpressing mice on a normal chow diet (Control n=6, Delta Gly n=3). Data are mean ±SEM.

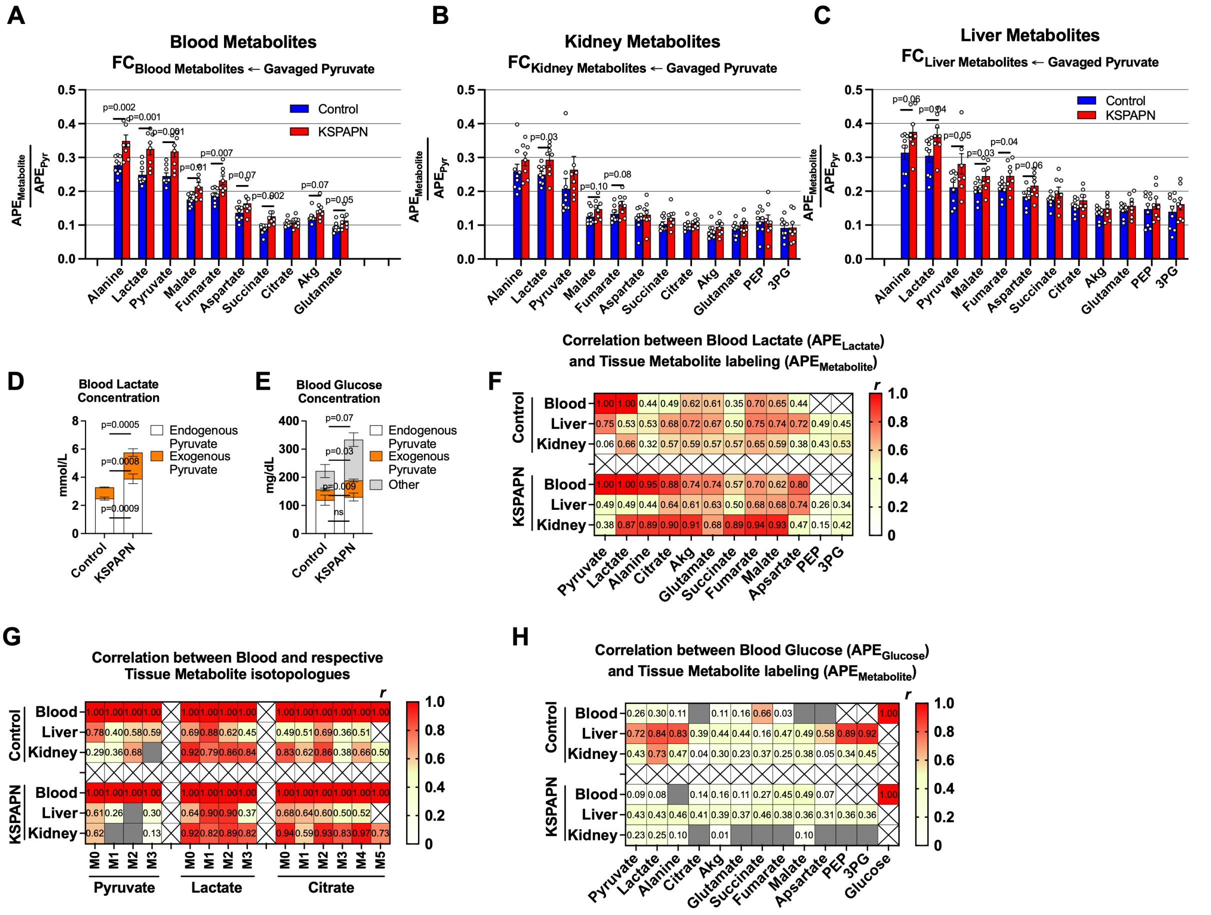

Figure S6. *Metabolite labeling from pyruvate in control and KSPAPN plasma 30 minutes after the [U-$^{13}C_3$]pyruvate gavage.*

(A-C) Fractional contribution (FC) of gavaged pyruvate to **(A)** blood, **(B)** kidney and **(C)** liver metabolites (Control n=9, KSPAPN n=8). **(D)** The contributions of exogenous (gavage) and endogenous pyruvate to blood lactate concentration (Control n=9, KSPAPN n=8). **(E)** The contributions of exogenous (gavaged) pyruvate, endogenous pyruvate, and other sources of carbon to blood glucose concentration (Control n=9, KSPAPN n=8). **(F)** Correlations between blood ^{13}C-lactate ($APE_{Lactate}$) and ^{13}C-metabolites ($APE_{Metabolite}$) in KSPAPN blood, kidney and liver (Control n=9, KSPAPN n=8). **(G)** Correlations between individual isotopologes of blood metabolites and their respective tissue isotopologes (e.g., correlation between blood lactate M+3 and either liver or kidney lactate M+3, etc) (Control n=9, KSPAPN n=8). **(H)** Correlations between blood ^{13}C-glucose ($APE_{Glucose\ C4\text{-}C6}$) and ^{13}C-metabolites ($APE_{Metabolite}$) in KSPAPN blood, kidney and liver (Control n=9, KSPAPN n=8). Data are reported as the mean ±SEM. Two-tailed unpaired Student's t test was used from (**A**) to (**E**).

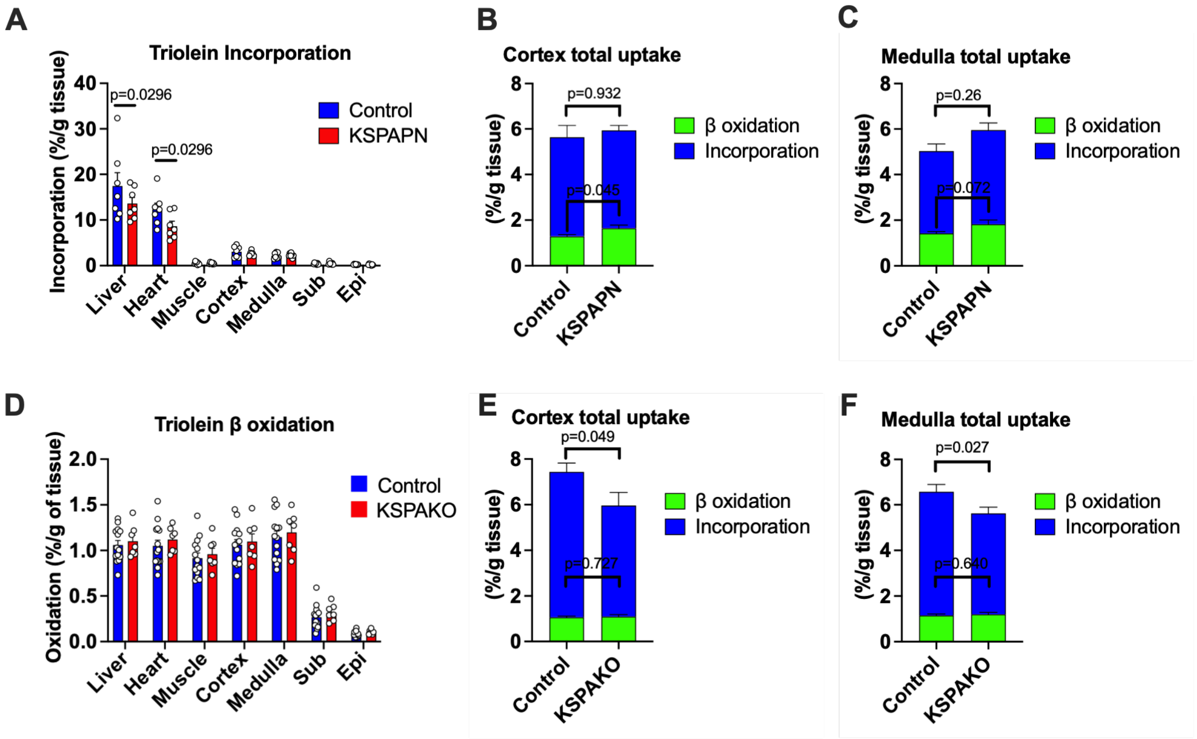

Figure S7. *[3]H-triolein uptake into KSPAPN and KSPAKO kidneys*
(**A**) ^{3}H-triolein lipid incorporation in KSPAPN tissues, including cortex and medulla, under chow diet conditions (Control n=7, KSPAPN n=7). Multiple unpaired two-tailed t-tests with 2-stage linear step-up procedure of BKY correction for multiple comparisons were performed to determine p-values. (**B**) The contribution of β oxidation and incorporation of ^{3}H-triolein to the total uptake in KSPAPN cortex (Control n=7, KSPAPN n=6). (**C**) The contribution of β oxidation and incorporation of ^{3}H-triolein to the total uptake in KSPAPN medulla (Control n=7, KSPAPN n=7). (**D**) ^{3}H-triolein lipid oxidation in KSPAKO tissues, including cortex and medulla, under chow diet conditions (Control n=14, KSPAKO n=7). (**E**) The contribution of β oxidation and incorporation of ^{3}H-triolein to the total uptake in KSPAKO cortex (Control n=14, KSPAKO n=7). (**F**) The contribution of *β* oxidation and incorporation of ^{3}H-triolein to the total uptake in KSPAKO medulla (Control n=14, KSPAKO n=7). Data are mean ±SEM. Unpaired two-tailed student's t-tests were performed for statistics for (**B**), (**C**), (**E**) and (**F**).

A

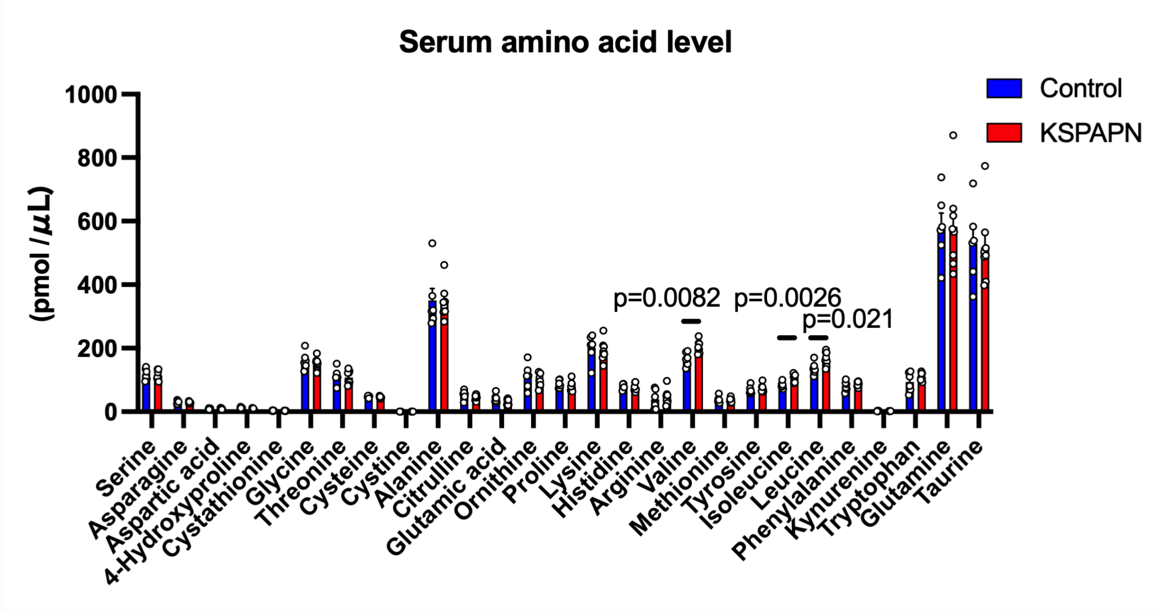

B

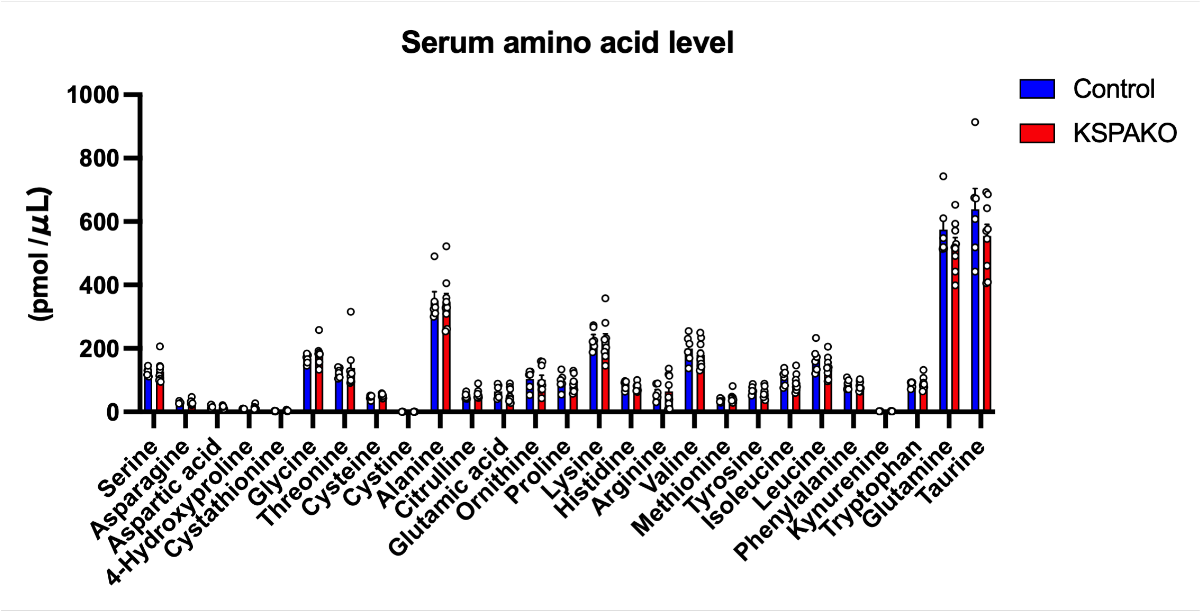

Figure S8. *Serum amino acid level in kidney specific adiponectin overexpression and kidney specific adiponectin KO mice under HFD.*

(A) Serum amino acid level in control and KSPAPN group were determined by mass spectrometry (Control n=6, KSPAPN n=8).

(B) Serum amino acid level in control and KSPAKO group were determined by mass spectrometry (Control n=6, KSPAKO n=8). Data are mean ±SEM. Multiple unpaired two-tailed t-tests with 2-stage linear step-up procedure of BKY correction for multiple comparisons were performed to determine p-values of **(A)** and **(B)**.

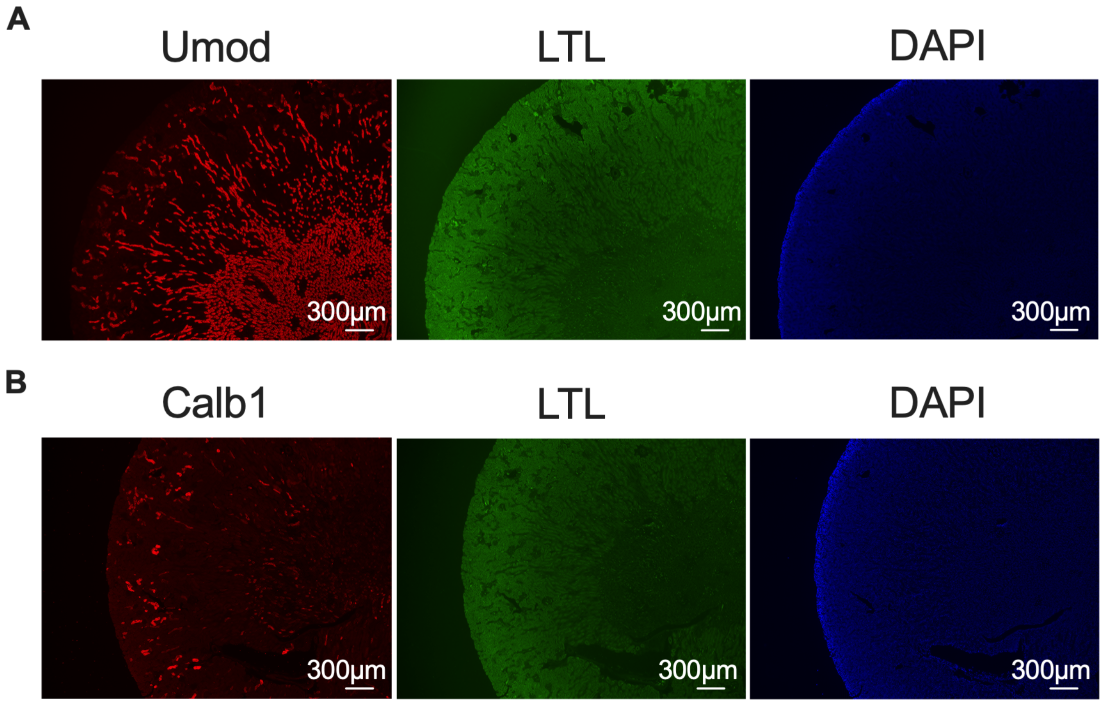

Figure S9. *Immunofluorescence of kidney tubular cell marker*
(A) Representative image of Immunofluorescence of WT kidney. Umod (red), LTL (green) and DAPI (blue). Scale bar indicates 300 µm. **(B)** Representative image of Immunofluorescence of WT kidney. Calb1 (red), LTL (green) and DAPI (blue). Scale bar indicates 300 µm.

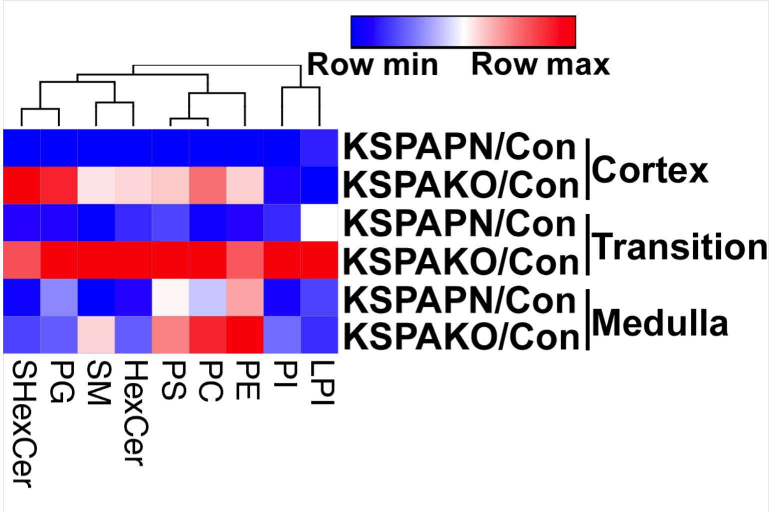

Figure S10. *The heatmap of the ratio of relative intensity of lipid species using a data base of lipids detected in human kidney tissue previously published in the literature and whose IDs have been confirmed by UHPLC-MS/MS.*(n=9)[58]

Table S1

List of genotyping primer sequences

Mouse strain	Forward primer	Reverse primer
KsprtTA	CCTAATCCAGCCTGTGAATGTAAGG	GCCGTTTATGACTTTGCTCTTGTC
TRE-Adiponectin	GACCACAATGGACTCTATG	CAAGGGACATCTTCCCATTC
Cre	GATTTCGACCAGGTTCGTTCACTCA	GCTAACCAGCGTTTTCGTTCTGCCA
Adiponectin flox	GGTGGCTCACAACCATTCATAA	CATACTCGCCTCTCCCAGAG
Alubumin Cre	ATGAAATGCGAGGTAAGTATGG	CGCCGCATAACCAGTGAAAC
Pepck flox	GCCCAGGATCTGCAGGGATGGACGG	ATAGGTGAGCTATGTCAAGGTT
Six2 Cre	ATGCTCATCCGGAGTTCCGTATG	CACCTTGTCGCCTTGCGTATAA
Glucagon receptor ko	GTCTTGTCGATCAGGATGATCTG	CAATATCACGGGTAGCCAACGC
Glucagon receptor wt	AGCTGGTCTGTAACAGAACC	CTGCTGGCTGCTATACATCT
Pparγ flox	TGTAATGGAAGGGCAAAAGG	TGGCTTCCAGTGCATAAGTT

Table S2

List of qPCR primer sequences

Gene name	Forward primer	Reverse primer
Pepck	CCACAGCTGCTGCAGAACA	GAAGGGTCGCATGGCAAA
G6Pase	TTACCAAGACTCCCAGGACTG	GAGCTGTTGCTGTAGTAGTCG
PGC1α	CAGCCTCTTTGCCCAGATCT	CCGCTAGCAAGTTTGCCTCA
Pcx	GCCTATGTGGAGGCTAACCA	CAGCTCTTCTGCCTGAGCTT
PPARα	GCCTGTCTGTCGGGATGT	GGCTTCGTGGATTCTCTTG
Timp1	CCCCAGAAATCAACGAGACCA	ACTCTTCACTGCGGTTCTGG
Collagen1a1	GCTCCTCTTAGGGGCCACT	CCACGTCTCACCATTGGGG
Collagen3a1	CTGGAGAACCTGGTGCAAAT	CCTCGGAAGCCACTAGGAC
Tgfβ1	ACCATGCCAACTTCTGTCTG	CGGGTTGTGTTGGTTGTAGA
Acta2	GTACCACCATGTACCCAGGC	GCTGGAAGGTAGACAGCGAA
Fn1	GCCCTGGTTTGTACCTGCTA	GGAATCTTTAGGGCGCTCAT
Emr1	TTACGATGGAATTCTCCTTGTATATCAT	CACAGCAGGAAGGTGGCTATG
Itgax	CTGAGAGCCCAGACGAAGACA	TGAGCTGCCCACGATAAGAG
Ccl2	CCACTCACCTGCTGCTACTCAT	TGGTGATCCTCTTGTAGCTCTCC
Mrc1	TGTGGTGAGCTGAAAGGTGA	CAGGTGTGGGCTCAGGTAGT
Tnfα	GGACAGTGACCTGGACTGTGG	AGTGAATTCGGAAAGCCCATT
Acc1	GAGGTACCGAAGTGGCATCC	GTGACCTGAGCGTGGGAGAA
Scd1	TGGGTTGGCTGCTTGTG	GCGTGGGCAGGATGAAG
Fas	CATCCACTCAGGTTCAGGTG	AGGTATGCTCGCTTCTCTGC
Srebp1c	GGAGCCATGGATTGCACATT	GCTTCCAGAGAGGAGGCCAG
Acly	CAGTCCCAAGTCCAAGATCCC	ACGATGGCCTTGGTATGTCG
PPARγ	GCCCAAACCTGATGGCATT	ATCTTAACTGCCGGATCCACAA
Cd36	GATGTGGAACCCATAACTGGATTCAC	GGTCCCAGTCTCATTTAGCCACAGTA
Lpl	CCGGAGAGACTCAGAAAAAGGTCATC	ACCCACTTTCAAACACCCAAACAAG
Abca1	TCCCAGAGCAAAAAGCGACT	GGCCACATCCACAACTGTCT
Fatp4	CGTTTCGACGGGTACCTCAA	ACATTCTCCCCTTTCCAGCG
Snap23	CAAACTACAGGAGCAGCCAGT	GAGCCATGTTCTTTAGGTTGCC
Vamp8	CCAGAATGTGGAGCGGATCT	CCTTCTGGGACGTTGTCTTGA
Abcg5	GCTAAATCACCCGATGTGCG	GGAAGTTTGCCGTGAATCTGG
Npc1l1	AGCTGAACTACGGAAGGTGC	GATGCCTGAGCGTATGTCCA
