# Supplementary material for: Endogenous renal adiponectin drives gluconeogenesis through enhancing pyruvate and fatty acid utilization
Source: Nat Commun. 2023 Oct 17;14:6531. doi: 10.1038/s41467-023-42188-4 (PMC10582045; doi:10.1038/s41467-023-42188-4)
Supplement: Supplementary file 3 — Description of Additional Supplementary Files [file 41467_2023_42188_MOESM3_ESM.pdf]

Description of Additional Supplementary Files

Title: Supplementary Data 1

Description: We semi-quantitatively analyzed lipid species distribution considering 17117 lipid ID species at 25 µm spatial resolution through the entire kidney section by IMAGEREVEAL™ MS software (Shimadzu Corporation).

We selected 3 areas of cortex, transition and medulla in each section based on the anatomical structure and compared "3 Control vs 3 KSPAPN" and "3 Control vs 3 KSPAKO".
