# Supplementary material for: Endogenous renal adiponectin drives gluconeogenesis through enhancing pyruvate and fatty acid utilization
Source: Nat Commun. 2023 Oct 17;14:6531. doi: 10.1038/s41467-023-42188-4 (PMC10582045; doi:10.1038/s41467-023-42188-4)
Supplement: Supplementary file 5 — Reporting Summary [file 41467_2023_42188_MOESM5_ESM.pdf]

nature portfolio

Corresponding author(s): Scherer Philipp

Last updated by author(s): 2023-9-12

Reporting Summary

Nature Portfolio wishes to improve the reproducibility of the work that we publish. This form provides structure for consistency and transparency in reporting. For further information on Nature Portfolio policies, see our Editorial Policies and the Editorial Policy Checklist.

Statistics

For all statistical analyses, confirm that the following items are present in the figure legend, table legend, main text, or Methods section.

n/a	Confirmed	
☐	☒	The exact sample size (*n*) for each experimental group/condition, given as a discrete number and unit of measurement
☐	☒	A statement on whether measurements were taken from distinct samples or whether the same sample was measured repeatedly
☐	☒	The statistical test(s) used AND whether they are one- or two-sided<br>*Only common tests should be described solely by name; describe more complex techniques in the Methods section.*
☐	☒	A description of all covariates tested
☐	☒	A description of any assumptions or corrections, such as tests of normality and adjustment for multiple comparisons
☐	☒	A full description of the statistical parameters including central tendency (e.g. means) or other basic estimates (e.g. regression coefficient) AND variation (e.g. standard deviation) or associated estimates of uncertainty (e.g. confidence intervals)
☐	☒	For null hypothesis testing, the test statistic (e.g. *F*, *t*, *r*) with confidence intervals, effect sizes, degrees of freedom and *P* value noted<br>*Give P values as exact values whenever suitable.*
☒	☐	For Bayesian analysis, information on the choice of priors and Markov chain Monte Carlo settings
☒	☐	For hierarchical and complex designs, identification of the appropriate level for tests and full reporting of outcomes
☒	☐	Estimates of effect sizes (e.g. Cohen's *d*, Pearson's *r*), indicating how they were calculated

Our web collection on statistics for biologists contains articles on many of the points above.

Software and code

Policy information about availability of computer code

Data collection	No software was used
Data analysis	Prism 10.0.2 software was used for plotting the data and statistics.

For manuscripts utilizing custom algorithms or software that are central to the research but not yet described in published literature, software must be made available to editors and reviewers. We strongly encourage code deposition in a community repository (e.g. GitHub). See the Nature Portfolio guidelines for submitting code & software for further information.

Data

Policy information about availability of data

All manuscripts must include a data availability statement. This statement should provide the following information, where applicable:

- Accession codes, unique identifiers, or web links for publicly available datasets
- A description of any restrictions on data availability
- For clinical datasets or third party data, please ensure that the statement adheres to our policy

All data supporting the findings of this study are available within the article and its supplementary materials, including Source Data. RNA sequencing data are available at NCBI GEO under the accession code (GSE242095)(https://www.ncbi.nlm.nih.gov/geo/query/acc.cgi?&acc=GSE242095). Raw Imaging mass spectrometry data is available in METASPACE (https://metaspace2020.eu/project/kidney_adipoq_ko) (https://metaspace2020.eu/project/kidney_adipoq_oe).

Research involving human participants, their data, or biological material

Policy information about studies with human participants or human data. See also policy information about sex, gender (identity/presentation), and sexual orientation and race, ethnicity and racism.

Reporting on sex and gender	n/a
Reporting on race, ethnicity, or other socially relevant groupings	n/a
Population characteristics	n/a
Recruitment	n/a
Ethics oversight	n/a

Note that full information on the approval of the study protocol must also be provided in the manuscript.

Field-specific reporting

Please select the one below that is the best fit for your research. If you are not sure, read the appropriate sections before making your selection.

[x] Life sciences [ ] Behavioural & social sciences [ ] Ecological, evolutionary & environmental sciences

For a reference copy of the document with all sections, see nature.com/documents/nr-reporting-summary-flat.pdf

Life sciences study design

All studies must disclose on these points even when the disclosure is negative.

Sample size	No statistical methods were used to determine sample size. Sample size was determined empirically or from sizes commonly published for the specific type of experiment (PMID:36696925, PMID36329217). We normally utilized 8 mice/group or more. We used less number of mice in case of revision because of the limited duration of time. For all mouse studies, the n value corresponds to individual mice of a given treatment. For in vitro-derived kidney cell culture experiments, the n values correspond to separate kidney cell preparations from separate mice or a different passage number of kidney from the same mouse.
Data exclusions	Statistical methods were used to identify and exclude outliers. For all Western blot densitometry, samples were removed from analysis if lanes contained artifacts, such as bubbles, which precluded accurate density calculations.
Replication	For mouse studies, key observations were confirmed more than twice independetly. For cell studies, we normally set up 3-4 replicates with independent experiments at least twice.
Randomization	All mice were randomly assigned to the control or experimental groups
Blinding	Complete Blinding was not implemented for the experiments in this paper because the mice are already tagged by ear punch and genotyped.

Reporting for specific materials, systems and methods

We require information from authors about some types of materials, experimental systems and methods used in many studies. Here, indicate whether each material, system or method listed is relevant to your study. If you are not sure if a list item applies to your research, read the appropriate section before selecting a response.

Materials & experimental systems

n/a	Involved in the study	
[ ]	[x]	Antibodies
[x]	[ ]	Eukaryotic cell lines
[x]	[ ]	Palaeontology and archaeology
[ ]	[x]	Animals and other organisms
[x]	[ ]	Clinical data
[x]	[ ]	Dual use research of concern
[x]	[ ]	Plants

Methods

n/a	Involved in the study	
[x]	[ ]	ChIP-seq
[x]	[ ]	Flow cytometry
[x]	[ ]	MRI-based neuroimaging

Antibodies

Antibodies used	[Primary Antibody]

	1:Rabbit anti-mouse polyclonal PCK1 antibodies (ab70358, Abcam) Dilution1:1000<br>2:Rabbit anti-mouse polyclonal Adipoq antibodies (produced in-house) Dilution1:1000<br>3:Mouse anti-mouse monoclonal α-tubulin antibodies (DM1A) (62204, Thermofisher) Dilution1:500<br>4:Rabbit anti-mouse monoclonal GAPDH antibodies (D16H11) (5174, Cell Signaling) Dilution1:1000<br>5:Rabbit anti-mouse Calbindin D28K Polyclonal Antibody (PA5-85669, Thermofisher) Dilution1:200<br>6:Rabbit Anti-mouse monoclonal UMOD antibody (EPR20071) (ab207170, abcam) Dilution1:1000<br>7:Lotus Tetragonolobus (Asparagus Pea) Lectin (LTL), fluorescein (FITC) (L32480, Thermofisher) Dilution1:100<br>[Secondary Antibody]<br>1:IR-Dye 800CW Donkey antibody against rabbit Li-Cor Cat#: 92632213 Dilution1:5000<br>2:IR-Dye 800CW Goat antibody against Mouse Li-Cor Cat#: 92632213 Dilution1:5000<br>3:IR-Dye 680RD Donkey antibody against Mouse Li-COR Cat:# 92668072 Dilution1:5000<br>4:Donkey Anti-rabbit Alexa fluor 594 (A-21207, Thermofisher)) Dilution1:500
Validation	[Primary Antibody]<br>1:Rabbit anti-mouse polyclonal PCK1 antibodies (ab70358, Abcam) (https://www.abcam.com/products/primary-antibodies/pck1pepc-antibody-ab70358.html)<br>2:Rabbit anti-mouse polyclonal Adipoq antibodies (produced in-house) (PMID:29224189)<br>3:Mouse anti-mouse monoclonal α-tubulin antibodies (DM1A) (62204, Thermofisher) (https://www.thermofisher.com/antibody/product/alpha-Tubulin-Antibody-clone-DM1A-Monoclonal/62204)<br>4:Rabbit anti-mouse monoclonal GAPDH antibodies (D16H11) (5174, Cell Signaling) (https://www.cellsignal.com/products/primary-antibodies/gapdh-d16h11-xp-rabbit-mab/5174)<br>5:Rabbit anti-mouse Calbindin D28K Polyclonal Antibody (PA5-85669, Thermofisher) (https://www.thermofisher.com/antibody/product/Calbindin-D28K-Antibody-Polyclonal/PA5-85669)<br>6:Rabbit Anti-mouse monoclonal UMOD antibody (EPR20071) (ab207170, abcam) (https://www.abcam.com/products/primary-antibodies/umod-antibody-epr20071-ab207170.html)<br>7:Lotus Tetragonolobus (Asparagus Pea) Lectin (LTL), fluorescein (FITC) (L32480, Thermofisher) (https://www.thermofisher.com/order/catalog/product/L32480)

Animals and other research organisms

Policy information about studies involving animals; ARRIVE guidelines recommended for reporting animal research, and Sex and Gender in Research

Laboratory animals	We generated two mouse models with doxycycline-inducible, kidney tubular cell-specific adiponectin overexpression or adiponectin KO mice by crossing KsprtTA mice with TRE-adiponectin or KsprtTA/adiponectin flox mice with TRE-Cre. All animals used in this study were littermate-controlled male mice and on a pure C57BL/6 background. Mice were maintained on a 12 h light/dark cycle in a temperature-controlled environment (22°C) and had free access to food and water. Water and cages were autoclaved. Cages were changed every other week, and the health status of the mice was monitoring using the Allentown sentinel filter, which started in the second quarter of 2017. The mouse genotype did not cause visible changes in initial weight, health or immune status. The age and number of the mice used for the experiments are indicated for each experiment in the Figure Legends. All of our experimental animals were kept under barrier conditions under constant veterinary supervision and did not display any signs of distress or pathological changes that warranted veterinary intervention. Mice were fed a standard rodent chow diet or 600 mg kg−1 doxycycline-containing chow or HFD diet containing or not 600mg-kg-1 doxycycline. In all cases where doxycycline was required to induce gene expression, it was supplied in the mouse diet. All mice received doxycycline diet, including controls (lacking the inducible transgenes (TRE-adiponectin or TRE-Cre).<br><br>The ages of mice that were used for experiments are as follows.<br>KsprtTA/TRE-adiponectin 10-50 weeks old.<br>KsprtTA/TRE-Cre/Adiponectin flox 10-50 weeks old.<br>Six2Cre/PPARγ flox 5weeks old<br>Albumin Cre/Pck1 flox 12weeks old<br>AdipoR2 KO 12 weeks old<br>ΔGly adiponectin overexpression 6-15 weeks old<br>Glucagon receptor KO 16 weeks old
Wild animals	The study did not involve wild animals.
Reporting on sex	In all experiments, only male mice were used, as female mice are more resistant to obesity and type 2 diabetes.
Field-collected samples	The study did not involve filed-collected samples.
Ethics oversight	All animal experimental protocols were approved by the Institutional Animal Care and Use Committee of University of Texas Southwestern (UTSW) Medical Center at Dallas, TX (APN# 2015–101207).

Note that full information on the approval of the study protocol must also be provided in the manuscript.
